# Muscle forces and the demands of human walking

**DOI:** 10.1242/bio.058595

**Published:** 2021-07-19

**Authors:** Adam D. Sylvester, Steven G. Lautzenheiser, Patricia Ann Kramer

**Affiliations:** 1 Center for Functional Anatomy and Evolution, The Johns Hopkins University School of Medicine, 1830 E. Monument Street, Baltimore, MD 21205, USA; 2 Department of Anthropology, University of Washington, Denny Hall, Seattle, WA 98195, USA; 3 Department of Anthropology, The University of Tennessee, Knoxville, Strong Hall, Knoxville, TN 37996, USA

**Keywords:** Musculoskeletal model, Biomechanics, Lower limb

## Abstract

Reconstructing the locomotor behavior of extinct animals depends on elucidating the principles that link behavior, function, and morphology, which can only be done using extant animals. Within the human lineage, the evolution of bipedalism represents a critical transition, and evaluating fossil hominins depends on understanding the relationship between lower limb forces and skeletal morphology in living humans. As a step toward that goal, here we use a musculoskeletal model to estimate forces in the lower limb muscles of ten individuals during walking. The purpose is to quantify the consistency, timing, and magnitude of these muscle forces during the stance phase of walking. We find that muscles which act to support or propel the body during walking demonstrate the greatest force magnitudes as well as the highest consistency in the shape of force curves among individuals. Muscles that generate moments in the same direction as, or orthogonal to, the ground reaction force show lower forces of greater variability. These data can be used to define the envelope of load cases that need to be examined in order to understand human lower limb skeletal load bearing.

## Abstract (precis)

**Summary:** A musculoskeletal model of human walking reveals the consistency, timing, and magnitude of lower limb muscle forces across the stance phase.

## INTRODUCTION

Reconstructing the locomotor behavior of extinct taxa is the attempt to reverse engineer an organism's behavior from a selection of its morphology (Sellers et al., 2005; Nyakatura et al., 2019). Paleontologists are typically limited to tissues that fossilize well (i.e. skeletal elements, teeth), and the endeavor is made more challenging by the fact that skeletons and skeletal elements are rarely complete (Sellers et al., 2005; Nyakatura et al., 2019). The mandate of this line of inquiry is to determine the ways in which the skeletal system reflects the demands of locomotion.

The mammalian locomotor skeleton can be viewed as a series of rigid levers that are connected at articulations and actuated by muscles. Skeletal elements allow muscles forces to be transmitted and applied to the environment/substrate, which produces animal motion. Skeletal elements must be able to withstand the structural demands of forces applied by muscles, adjacent skeletal elements, and the substrate (Martin et al., 2015). Thus at a basic level, the skeletal system reflects the forces associated with locomotion. Such form-function relationships are informed by studying extant organisms, and then extinct taxa can be understood by projecting principles into the past. That is, reconstructing past behaviors requires evaluating the skeletal performance of extinct animals, which in turn necessitates discerning the principles that link behavior (e.g. walking), function (e.g. forces and load bearing), and morphology (e.g. femoral shape) using extant animals.

As bipedalism is one of the foundational transitions in human evolution, understanding the adaptive origin of hominin bipedalism remains a critical task and one that ultimately depends on elucidating the details of earlier forms. Based on fossilized skeletal material, various researchers have argued that most, if not all, extinct hominins practiced forms of bipedalism that were kinematically, kinetically, and/or metabolically distinct from modern human bipedalism (e.g. Stern and Susman, 1983; Kramer, 1999; Wood and Collard, 1999; Simpson et al., 2008; Lovejoy et al., 2009; Been et al., 2012; Ruff and Higgins, 2013; DeSilva et al., 2013). Much of this research has focused on the femur and pelvis because of the critical role these structures play in weight-bearing and hominin locomotion, and these bones contain features that are universally agreed to indicate bipedalism, while also potentially revealing the uniqueness of earlier forms (e.g. femoral bicondylar angle, Johanson and Taieb, 1976; femoral neck cortical distribution, Ruff and Higgins, 2013; short and wide pelvis, Lovejoy et al., 2009).

Leveraging a model adopted from human orthopaedic biomechanics, Lovejoy and colleagues (Heiple and Lovejoy, 1971; Lovejoy et al., 1973) were the first to estimate forces exerted on the proximal femur of early hominins during walking (Ruff, 2018). This highly influential work modelled the midstance of walking based on standing on one foot as established by Frankel and Burstein (1970) and also developed by McLeish and Charnley (1970). This model has been used repeatedly to evaluate the structural capacity of early hominin lower limb skeletal elements (e.g. Berge, 1994; Ruff, 1998). For example, the human femoral neck is unique relative to other apes in its superoinferior asymmetric distribution of cortical bone (Lovejoy, 1988; Lovejoy et al., 2002). From these principles, Ruff and Higgins (2013) argue the South African hominins *Australopithecus africanus* and *Paranthropus robustus* would have utilized a bipedal gait that required lateral sway of the trunk.

Without diminishing the importance and influence of this body of work, the limitations of this approach, although state-of-the-art at the time, should be recognized. As with all models, the estimates of forces were generated under a specific set of simplifications (assumptions) to make the problem tractable. Implicit in the single limb standing model is that midstance is sufficiently representative of the entire stance phase of the gait cycle to describe the structural demands impose by lower limb biomechanics. During standing, however, the ground reaction force (GRF) is equal to body weight, while at walking midstance, the GRF is closer to 70% of body weight because the center of mass (CoM) of the body has been accelerated upward (Richards, 2008). Second, during single limb standing, the total body CoM must be directly above the support foot in order to maintain balance, but this is not the case for walking (Lugade et al., 2011). Additionally, the GRF magnitude fluctuates during walking stance phase and is lower at midstance than at other points in the gait cycle (Richards, 2008). Finally, this simplified approach poses the question of walking biomechanics as a two-dimensional coronal plane problem. While standing is a static condition in which body weight is reacted vertically by the ground with only minimal horizontal plane reaction forces needed for stabilization, walking is dynamic and the ground reaction resultant must include the anteroposterior components required to brake and propel the body in the direction of progression, a sagittal plane problem. Although these simplifications were perhaps necessary at the time, walking is inherently three-dimensional in terms of space with an additional time dimension. Critical information is potentially lost by projecting this four dimensional problem into two dimensions. Collectively, these limitations argue that forces during walking cannot be adequately modeled by single limb standing and suggest that early hominin skeletal elements should be reexamined with the aid of more complete internal force (e.g. muscle) estimates.

Measuring muscle forces in living animals is exceptionally challenging because it generally requires surgical intervention to implant the relevant sensors into the body (Bey and Derwin, 2012; Pedersen et al., 1997). As an additional obstacle, these techniques are normally limited to a single joint or muscle/tendon within a single study (see Bey and Derwin, 2012 for examples). Thus, directly measuring muscle force at the level of individual muscles during activity is challenging at best, if not beyond current technology. Mechanical calculations have also been used (i.e. inverse dynamics, net joint moments), but these do not account for essential components of muscle activity (e.g. allocating moments among muscles, isometric contractions, antagonistic muscle activity) and hence also fail to provide a complete picture. The current methodology that bridges the gaps between morphology and gait mechanics is musculoskeletal modeling. Despite its widespread use in the fields of orthopaedics, human biomechanics, and biomedical engineering, its application to inform paleontological locomotor reconstructions is relatively underdeveloped (but see, Bishop et al., 2021; Hutchinson et al., 2005; Nagano et al., 2005; Sellers et al., 2005, 2009; Wang et al., 2004 for examples).

Musculoskeletal modeling has emerged as an important technique for analyzing the details of human (and other animals) movement (e.g. Delp et al., 2007; O'Neill et al., 2013). Musculoskeletal models build upon forward and inverse dynamic rigid-segment models that represent the body as a series of segments (e.g. the thigh), which are linked together at frictionless, undeformable connections (i.e. joints). In inverse dynamic analyses, a critical issue, known as the muscle redundancy problem, is determining how to apportion the net joint moments to particular muscles (Anderson and Pandy, 2001; Stanev and Moustakas, 2019). Some attempts to solve this issue have apportioned force based on simple assumptions of uniform muscle activation (i.e. distributed based on physiological cross-sectional area; e.g. Perry et al., 2011; Warrener et al., 2015). This assumption is, however, not well supported (Crowninshield and Brand, 1981). Musculoskeletal models include models of individual muscles (or even portions of muscles), which are used to solve the muscle redundancy problem by minimizing a particular criterion. Frequently, the minimization metric is the sum of muscle activations raised to a power (Anderson and Pandy, 2001). Additionally, musculoskeletal modeling can account for the coactivation of antagonist muscles and biarticular muscles.

Results of modeling simulations can reveal the activations of individual muscles, as well as estimates of joint contact and muscle forces (Anderson and Pandy, 2001; De Pieri et al., 2018; Delp et al., 2007). The availability of several software packages (e.g. AnyBody Modeling System, OpenSim, Freebody) has lowered barriers to musculoskeletal modeling, and models are now routinely used in human gait research to estimate muscle activity, muscle force, muscle moment arms, joint contact forces, and a host of other potential variables. Much of this work has reported aspects of model development (Anderson and Pandy, 2001; Arnold et al., 2010; Carbone et al., 2015; Delp et al., 1990) and examined the sensitivity of model simulations to a number of different modeling choices (e.g. muscle parameters; Ackland et al., 2012). Researchers have also leveraged musculoskeletal modeling to answer specific questions, presenting results for those muscles influencing their particular question (e.g. knee contact forces and muscles that cross the knee; Besier et al., 2009; Fraysse et al., 2009). Often these investigations have a specific goal of understanding a pathological mechanism or recovery from it (e.g. Komura et al., 2004). Liu et al. (2008) report body mass-scaled muscle forces for muscles of the lower limb during walking for eight subjects that had an average age of 12.9 years old (range 7–18 years old). Thus, a complete reporting of lower limb muscle forces during typical human walking for a range of healthy adult humans appears lacking in the literature (but see Skubich and Piszczatowski, 2019 for thigh musculature). Such a data set is crucial for delineating the demands on the human skeleton and future investigations of early hominins.

Our ultimate goal is to evaluate skeletal performance and reconstruct locomotor behaviors in extinct hominins, grounded in an understanding of human skeletal performance during locomotion. The first step toward that goal is to use a musculoskeletal model to identify muscles that exert substantial forces on lower limb skeletal elements during walking. To that end, we seek to determine which muscles produce consistent patterns of force generation among humans over multiple steps and which muscle force profiles are more variable. As a complementary goal, we seek to determine the magnitude of lower limb muscle forces during walking and when during the gait cycle peak forces occur. This information will allow us to define the envelope of load cases that must be explored to examine skeletal performance during bipedal walking.

Decades of human locomotor research (Inman et al., 1981; Perry and Burnfield, 2010; Winter, 1991) allows us to generate predictions with regards to muscles group functionality in bipedalism. We predict that the principal requirements for human bipedalism of support and propulsion will generate the largest, and most consistent, muscle forces. These muscle functional groups include the triceps surae complex during toe off, the hip abductor mechanism for pelvic support during single support phase, and the knee extensor group for preventing knee collapse during stance phase (Perry and Burnfield, 2010; Webster and Darter, 2019; Winter, 1991). We also predict that muscles which generate joint moments in the same direction as, or orthogonal to, the GRF will be more variable in their pattern of force generation and smaller in magnitude. For example, the hip adductors, which generate hip moments in the same direction as the GRF, will be more variable and forces will be smaller in magnitude than hip abductors. We predict that this will also be true of the external hip rotators, which act largely in a plane orthogonal to the GRF.

## RESULTS

**Fig. 1.**
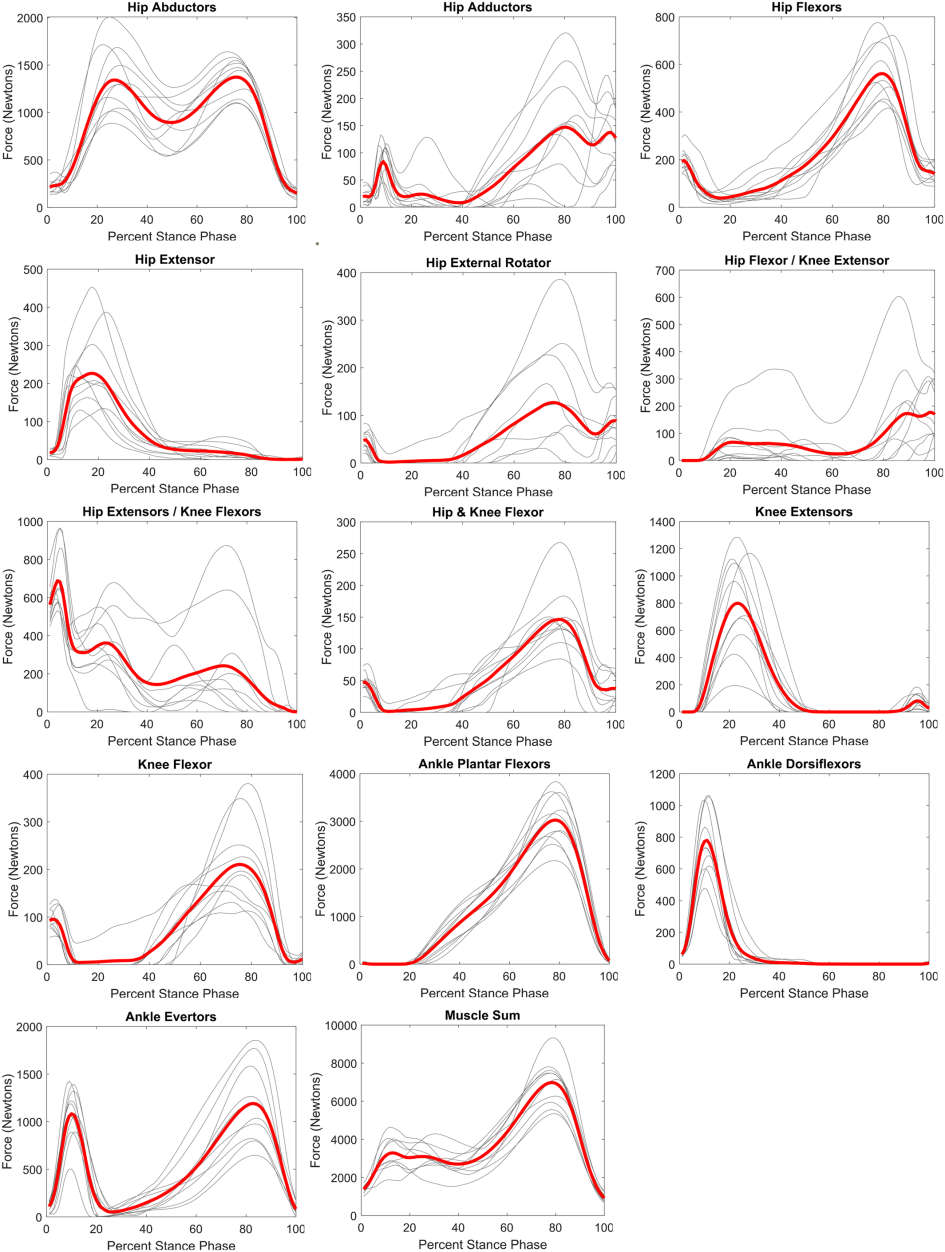
**Functional group muscle force profiles during walking stance phase.** Grey lines are average curve for each participant (six to ten stance phases per participant). Red lines are average of the ten participant average curves.

The Muscle Sum curve exhibits a large peak late in the stance phase (78% of stance phase), and two smaller closely timed peaks early in stance phase (either side of ∼20% of stance) (Fig. 1) for all participants (Table 1). The early and late force peaks are separated by a valley at 40% of stance phase. Several functional muscle groups exhibit consistent timing and shape of the force curve across participants (Fig. 1; Table 2). The ankle plantar flexor group (including gastrocnemius medialis, gastrocnemius lateralis, soleus) have the highest correlation (r=0.98), showing an exceptionally high consistency in the timing and pattern of force generation by this muscle group during the stance phase of walking (Fig. 1; Table 2). This muscle group also shows the highest average force peak (3025N) at 78% of stance phase. The hip adductor (r=0.70), hip external rotators (r=0.58), hip extensor/knee flexor (r=0.70), and hip flexor/knee extensor (r=0.55) muscle groups show distinctly lower correlations compared to the remaining nine muscle groups (r≥0.88) (Table 2).

**Table 1.**
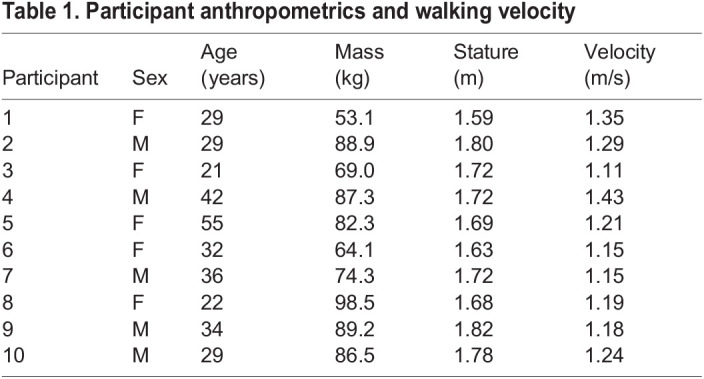
Participant anthropometrics and walking velocity

**Table 2.**
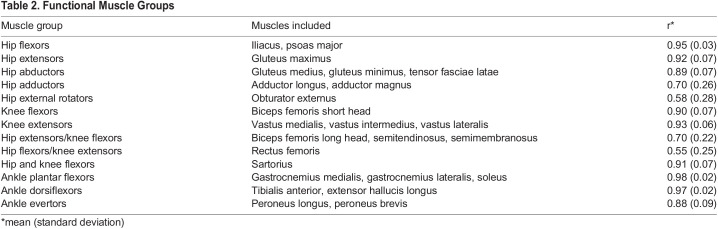
Functional Muscle Groups

**Fig. 2.**
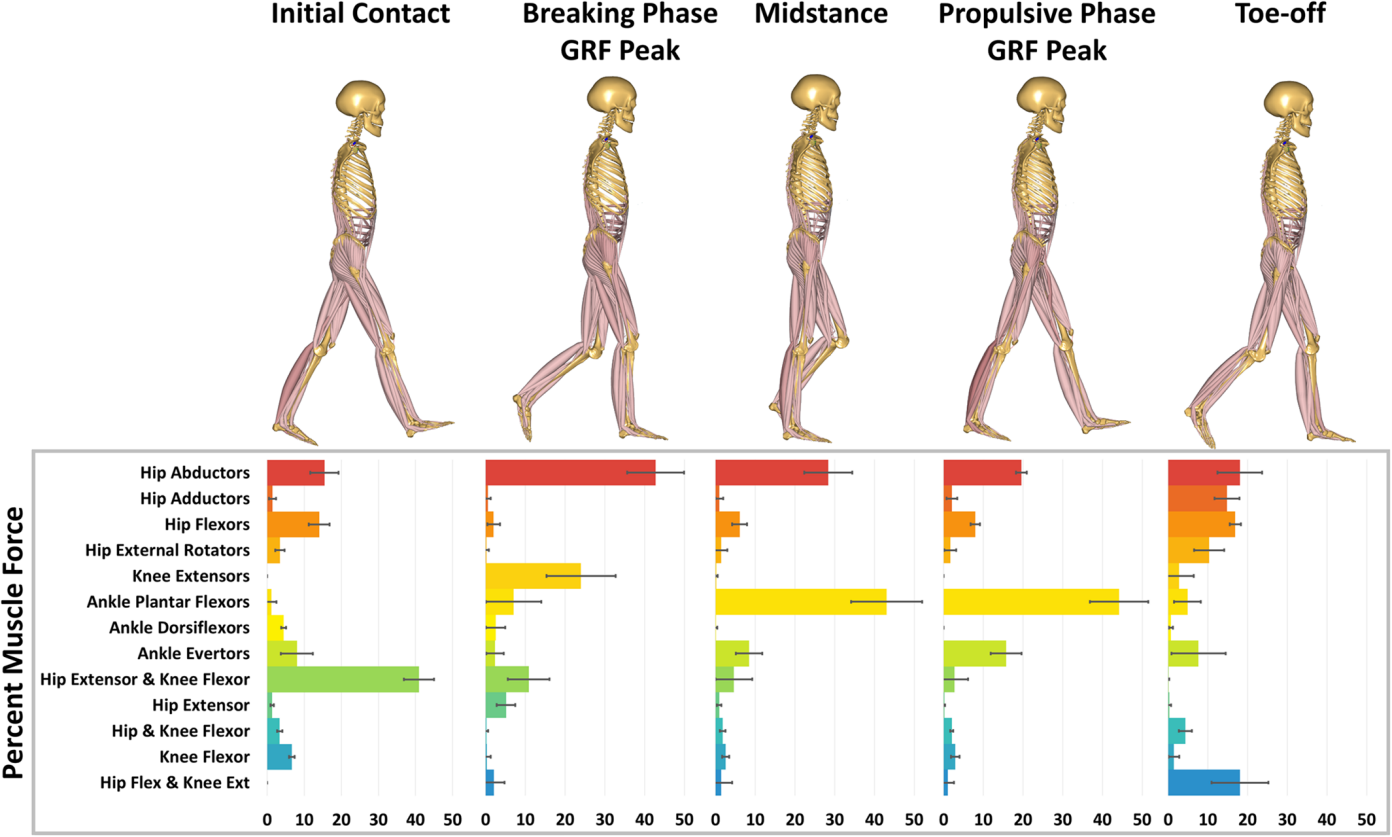
**Muscle force expressed as a percentage of the Muscle Sum.** Five specific gait events: initial contact; peak in the vertical GRF during the breaking phase of stance; midstance; peak in the vertical GRF during the propulsive phase of stance; toe-off.

At initial contact, the hip extensor/knee flexor group shows both the greatest percent muscle force (Fig. 2), but overall force production at initial contact is low compared to subsequent gait events. The second gait event is the breaking phase GRF peak (26% of stance phase). At this time interval the hip abductor group generates the greatest percentage of force, followed by the knee extensor group. During midstance (50% of stance phase) and propulsive phase GRF peak (78% of stance phase), the hip abductors and the ankle plantar flexors generate the greatest percentage of the total force generated.

**Fig. 3.**
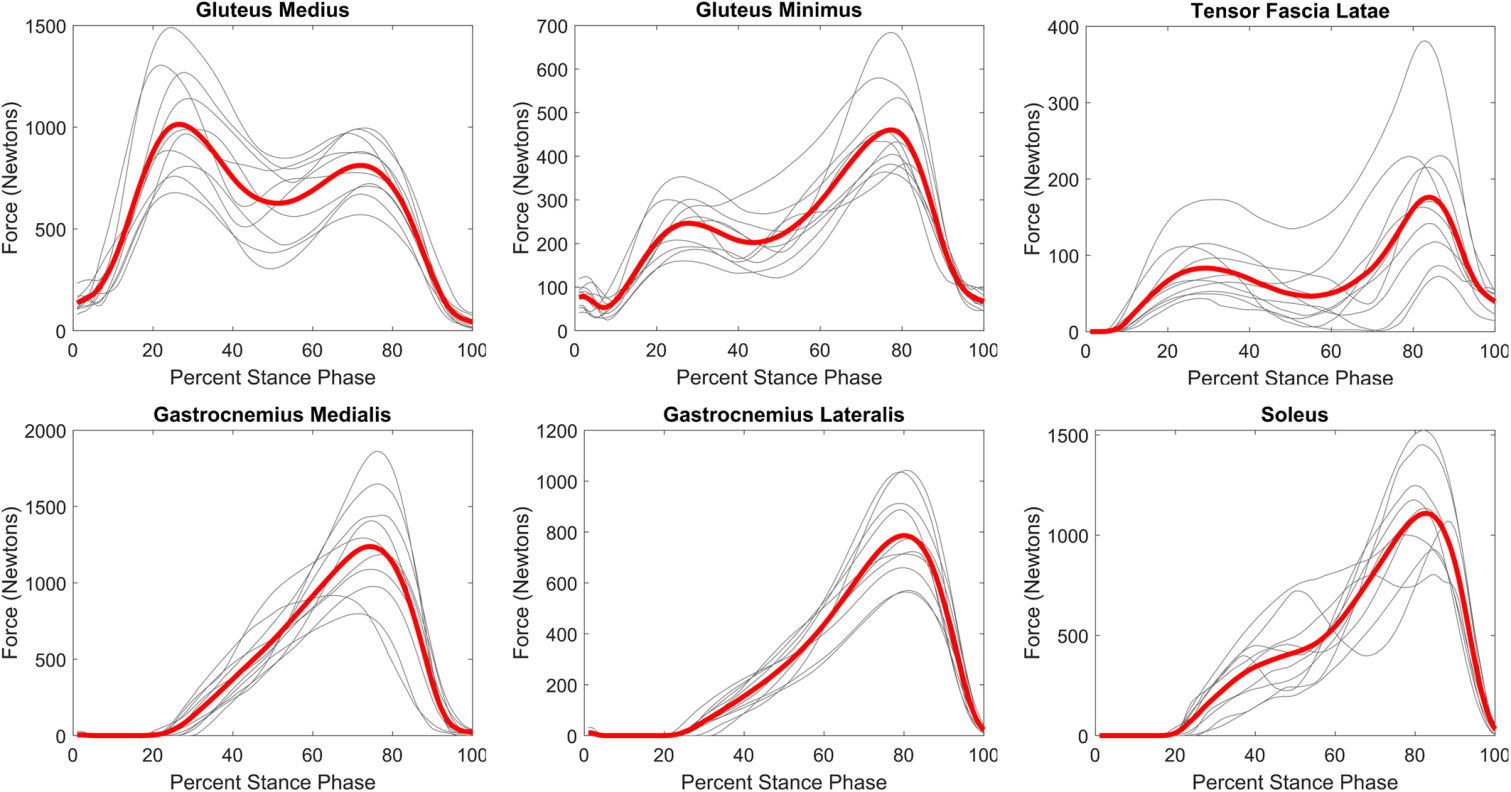
**Muscle force profiles for anatomically defined muscles during walking stance phase.** Grey lines are average curve for each participant (six to ten stance phases per participant). Red line is average of the ten participant average curves.

**Fig. 4.**
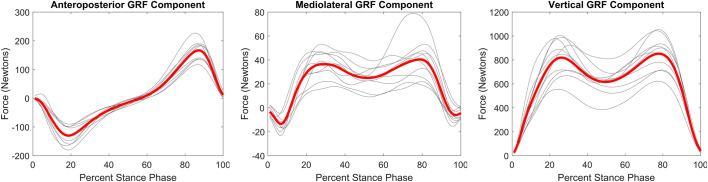
**Ground reaction force profiles during stance phase.** Grey lines are average curve for each participant (six to ten stance phases per participant). Red line is average of the ten participant average curves.

All three hip abductor muscles evince a curve with two peaks that are temporally aligned with the peaks in the GRF (Figs 3 and 4). Within the ankle plantar flexors, all muscles demonstrate the same basic shape of the force curve in which the muscle becomes active at around 20–30% of stance phase and peaks between 75–85% of stance phase (Fig. 3). The average gastrocnemius medialis and soleus peaks are within 100N of each other (1238N versus 1109N) and generate more force than gastrocnemius lateralis (peak=786N).

## DISCUSSION

Supporting literature-based predictions (Perry and Burnfield, 2010; Webster and Darter, 2019; Winter, 1991), muscles that act to maintain support or generate propulsion evinced the most consistent shape of their muscle force curves compared to other muscle groups. This result is encouraging because the muscle redundancy problem means that many modeling solutions to muscle force allocation are potentially capable of producing the desired motions. The results presented here, however, demonstrate that these musculoskeletal simulations produce activity in functional groups that are typically described as critical for human bipedalism (Perry and Burnfield, 2010) and agree with thigh muscle force curves reported by Skubich and Piszczatowski (2019). The two functional muscle groups with the highest consistency in shape and timing of force are the ankle plantar and dorsiflexors. This is perhaps expected given the consistently close proximity of the GRF force vector to the ankle. The ankle dorsiflexors are active in the early portion of the stance phase when the GRF passes posterior to the ankle, and these muscles control the descent of the foot to the floor/substrate (Perry and Burnfield, 2010; Webster and Darter, 2019). The GRF quickly passes anterior to the ankle joint at which time the ankle plantar flexors maintain the position of the ankle, and then in the later portion of stance, contract to propel the body forward and up (Perry and Burnfield, 2010; Webster and Darter, 2019). The ankle plantar flexors also generated the largest magnitude force of any functional muscle group, consistent with the critical propulsion task. A similar relationship can be seen in the hip extensors and flexors as a function of the GRF position relative to the hip joint. The hip extensors are active following initial contact when the GRF passes anterior to the hip, while the hip flexors are active later in stance as the GRF passes posterior to the hip (Perry and Burnfield, 2010; Webster and Darter, 2019).

Given the high consistency of force production for most functional muscle groups, it is interesting that four functional muscle groups showed significantly higher variation in the shape and timing of their force curves. These are the hip adductors, external hip rotators, hip flexor/knee extensor, and hip extensor/knee flexor groups. The hip adductors act to generate moments in the same direction as the GRF, while the external hip rotators act in a plane roughly orthogonal to the GRF, suggesting these muscle groups are driven by requirements to provide finer control of hip stability (i.e. tweak hip moments). The two other muscle groups with lower consistency in their muscle force curve shape are hip flexor and knee flexor group (i.e. rectus femoris) and the hip extensor and knee flexor group (i.e. biceps femoris long head, semitendinosus, semimembranosus). It is important to note that all four of these muscles have proximal attachments on the pelvis and distal attachments on the tibia and are capable of producing forces that act in parasagittal plane movement. These muscles are biarticular, and it may be that these muscles are not as critical for level-ground bipedal walking, but rather are more important for other terrains or tasks. Alternatively, the activity of these muscle may reflect segment kinematics that are difficult to capture with high fidelity. In general, segment kinematics in the sagittal plane tend to present consistent patterns across individuals and trials (Fig. S1). This is not true, however, for pelvic tilt, which evinces a kinematic pattern that is variable across individuals and trials (see Fig. S1 for accelerations that demonstrate this phenomenon more apparently). This variability may be the product of limitations in marker based kinematic data collection (Cereatti et al., 2017; Fiorentino et al., 2020; Leardini et al., 2005), and thus the variable muscle activity could reflect noise inherent to the data collection and modelling processes and not a natural phenomenon of human walking. Alternatively, the variation in pelvic tilt among steps or people may be a normal aspect of walking as the body adjusts to accommodate variation in the location of the center of pressure of the ground reaction force (Lugade and Kaufman, 2014), or controlling trunk motion (Schumacher et al., 2019). Finally, unlike propulsion or coronal plane stability, knee stability is accomplished via coordination of the hip, knee and ankle joint positions and moments (Winter, 1991), allowing flexibility in individual joint patterns that manifests in this analysis as inconsistency. Future work should examine the muscles with high force magnitude and inconsistent patterns to investigate the causes of pattern inconsistency.

In addition to the highly consistent force curve shape, the hip abductor, ankle plantar flexor, and knee extensor muscle groups generate the largest muscle forces in the lower limb during walking. Other muscles represent larger percentages at initial contact (hip extensor and knee flexors) and toe-off (hip flexor and knee extensor); however, the Muscle Sum at these two gait events are substantially smaller than during the two GRF peaks and midstance. At the early GRF peak, the knee extensors and hip abductors account for more than half of the total force production, consistent with their roles in counteracting the GRF to prevent knee collapse and support the pelvis in the coronal plane (Perry and Burnfield, 2010; Webster and Darter, 2019). During midstance, the ankle plantar flexors represent the largest muscle force, while hip abductors continue to incur the second largest, again consistent with well-established roles of these muscles (Perry and Burnfield, 2010; Webster and Darter, 2019). During the second GRF peak, which also represented the greatest force production, the ankle plantar flexors generate approximately twice as much force as any other muscle group, supporting their critical function in human walking of propelling body mass. Collectively, these results suggest that to evaluate the structural capacity of the lower limb, the late stance propulsion peak presents the greatest structural demand on the lower limb skeleton, followed by the early stance braking peak.

### Muscle activity within hip abductor and ankle plantar flexor groups

The musculoskeletal model reveals a more detailed understanding of force production within hip abductor and ankle plantar flexor groups. Within the ankle plantar flexor group, gastrocnemius medialis and soleus produce similar muscular force contribution to generate ankle moments during walking, while gastrocnemius lateralis makes a slightly smaller contribution. The hip abductor muscle force curves show activity patterns in different muscles that are expected based on the position of muscles and fiber orientation relative to the GRF. Gluteus medius generates more force than the other two muscles in the hip abductor category, approximately two to three times that of gluteus minimus throughout the stance phase. Because gluteus medius is a larger muscle with a larger lever arm, it should carry more of the burden (Klein Horsman et al., 2007; Németh and Ohlsén, 1989). Additionally, it is noteworthy that gluteus minimus generates more force in the second half of the stance phase, when the hip is in a more extended position, while gluteus medius makes a larger contribution in the first half of stance phase when the hip is in a flexed position. The majority of the gluteus medius origin is positioned posteriorly to the hip with fibers running anteriorly and inferiorly to the proximal femur. Thus, the muscle fibers of gluteus medius are more aligned with the direction of the GRF during the first half of stance phase. The gluteus minimus origin is more anteriorly located with fibers more aligned to the GRF later in stance phase, accounting for its great activation during the second half of stance.

### Hominin evolution

Adaptations to bipedal walking and running have been previously demonstrated in the human ankle plantar flexor muscles compared to the same muscles in non-human great apes. The human gastrocnemius muscle is short, pennated, and attached to a long Achilles tendon, whereas non-human great apes have long muscle bellies with more parallel fiber orientations (Aerts et al., 2018). The long human Achilles tendon is thought to be an energy storage device that reduces the metabolic cost of running (Bramble and Lieberman, 2004), while the long muscle bellies in the non-human apes allow for a larger range of motion (Aerts et al., 2018). Additionally, calcaneal tuber length represents the lever arms for the triceps surae, dictating the force required to generate a given plantarflexion moment. Relative to body mass, humans have longer posterior calcaneus (proxy for the triceps surae lever arm) than the non-human great apes (Harper, 2020). The shorter non-human ape posterior calcaneus enhances range of motion, while the relatively long human posterior calcaneus enhances the generation of plantar flexion moments. These adaptations in the human triceps surae complex (i.e. muscle, tendon, calcaneus) are consistent with the important role of this muscle demonstrated here using the musculoskeletal model.

Aspects of morphology that affect the hip abductor mechanism, have long been examined to evaluate relative bipedal capability and metabolic efficiency among modern humans (e.g. Warrener et al., 2015) and across the human lineage (e.g. Lovejoy et al., 1973; Ruff, 1995; Vidal-Cordasco et al., 2017). In an early biomechanical analysis, Lovejoy et al. (1973) argue that dimensions of the pelvis and proximal femur indicate early hominins would have experienced hip joint reaction forces during walking that produced similar hip joint pressures compared to modern humans. Although muscle forces were not presented, similar joint reaction forces must reflect proportionally similar hip abductor forces. Based on a similar biomechanical model, Ruff (1995) argued that *Homo erectus* (and other erectus-like specimens) would have required relatively greater hip abductor forces during walking. Ruff (1998) and Ruff and Higgins (2013) suggest that, based on femoral morphology, the earlier australopiths would have utilized a bipedalism that included lateral trunk sway. These previous analyses of the hip abductor mechanism have evaluated abductor muscle forces at walking midstance or a midstance proxy (i.e. single limb standing) (Lovejoy et al., 1973; Ruff, 1995; Warrener et al., 2015). Our results demonstrate, however, that the force generated by the hip abductors are not greatest at midstance, arguing it may be additionally profitable to examine the hip abductor force peaks.

Beyond structural concerns of the proximal femur, the hip abductor mechanism has also been implicated as a source of metabolic cost during human walking (Christopher, 2017; Warrener et al., 2015). Without including information about muscle length, activation and lengthening/shortening (Koelewijn et al., 2019), it is potentially problematic to interpret muscle force as a direct proxy for metabolic cost. Higher force production or longer activation of muscle tissue does increase the energy consumption of muscle tissue (e.g. Ortega et al., 2015), but the relationship between force and energy consumption is complex. Those caveats aside, the fluctuation of the both the ankle plantar flexors and hip abductors argues that their metabolic demands may also fluctuate across stance phase. Consequently, isolating the metabolic consequences of one muscle group by looking at its activity at one time point in the gait cycle is likely difficult (e.g. Warrener et al., 2015). This difficulty is further complicated by the fact that metabolic energy consumption (as measured by the volumetric rate of oxygen consumption) cannot be collected or expressed at that level of temporal resolution, requiring that such analyses look for correlations that are sufficiently strong to be detectable across the gait cycle (e.g. Vidal-Cordasco et al., 2017).

While first principles may lead to expectations regarding morphology (e.g. lever arms) and metabolic costs, a significant, potentially insurmountable, challenge exists in attempting to connect variation in modern human musculoskeletal morphology with metabolic cost. The pelvis, perhaps, provides the clearest example. Researchers have long argued that modern human pelvic morphology reflects two competing selective pressures: obstetrics and locomotion (e.g. Warrener et al., 2015; Washburn, 1960). Selection for obstetrics should favor a wider pelvis (i.e. larger birth canal) because it acts to decrease infant and maternal mortality (Mitteroecker et al., 2016). Conversely, narrower pelves (especially bi-acetabular breadth) should accrue metabolic saving because narrower pelves require lower hip abductor forces to stabilize the pelvis (Warrener et al., 2015), and the abductors are a contributor to cost. Metabolic savings should increase fitness because energy not allocated to locomotion can be invested in offspring. Under such a regime of strong stabilizing selection, both wide and narrow pelves result in lower fitness. The result would be that living modern humans represent a range of pelvic variation which has small metabolic consequences, especially when considered in the context of other aspects of locomotor morphology (Vidal-Cordasco et al., 2017). Further, the metabolic variation that can be attributed to pelvic morphology may be obscured by other sources of variation (e.g. physiological processes) and thus impossible to detect in a laboratory setting. For that reason, attempts to understand relationships between skeletal morphology and metabolic energy consumption using only the variation present in living modern humans may be, at best, misdirected and, at worst, futile.

It is worth acknowledging that some humans are more economical and effective bipeds compared to other humans (e.g. world class marathon runners). Those individuals, however, exist as a package of morphology and physiology, making it impossible to isolate the effect of a particular variable of interest (e.g. bi-acetabular breadth) from other potentially important variables (e.g. body composition, lung volume, heart stroke volume, lactic acid tolerance, muscle architecture, metabolic economy). The results presented here suggest the relationship between hip abductors and metabolic cost should be revisited using alternative approaches that minimize the variation introduced by physiology. Specifically, efforts to understand morphology, especially those regions under strong selection, could benefit from mechanical analyses.

### Limitations

As with all modeling exercises, input parameters, including uncertainty in motion tracking marker placement, movement artifacts, and body segment parameters, may affect modeling results (Lamberto et al., 2017; Myers et al., 2015). Additionally, subjects chose their normal walking velocity, and we did not investigate slower or faster movements, inclined or declined walking, other bipedal behaviors (e.g. running), or terrain complexity. All of these experimental choices may limit the inferences regarding the generated muscles forces. Human musculoskeletal models, such as the one employed here, rely on measurements of muscle morphology (e.g. volume, pennation angle) to parameterize muscle element models. The muscle morphology parameters used in this model have been used in many studies (e.g. Fluit et al., 2014; Lund et al., 2015; Modenese and Phillips, 2012; Trepczynski et al., 2012), but they derive from a small sample of cadavers that were elderly at the time of death (Klein Horsman et al., 2007). Because muscle force allocation is based in minimizing the cube of muscle activation, small variation in particular muscle parameters may have large effects on muscle force and muscle force allocation and more examination of the sensitivity of the model results to muscle morphology (particularly of younger people) is warranted. Myers et al. (2015) found muscle forces to be particularly sensitive to tendon slack length. Tendon slack length, which is a parameter of the Hill-type muscle model, has a direct influence on the region of the force-length curve in which a muscle operates. The muscle models use here, however, generate force only as a function of activation and maximum isometric contraction force. Our use of a simpler muscle model is preferable because it reduces the number of model parameters (and hence complexity of muscle sensitivity) and is appropriate for motions such as walking during which muscles contract at slow velocities near the peak of their force-length curves (Fischer et al., 2018). Nonetheless, additional efforts are required to understand the sensitivity of model outputs to all input parameters.

### Conclusion

Musculoskeletal modeling was used to clarify the details of muscle force generation during walking. Using the kinematic and kinetic data from ten participants, we found patterns that confirm general predictions based on decades of research. Muscles that contribute to support and propulsion display the greatest consistency in the timing and shape of force generation. This is consistent with their roles as the main drivers of human walking. Muscles that act to generate moments in the same direction as GRF-generated moments, or in planes perpendicular to the GRF, show considerably less consistency in the shape and timing of force generation, and are generally much lower in magnitude. This is consistent with their roles as stabilizers of walking and as muscles that make minor modifications to joint moments. In extending our understanding across the entire stance phase we determined that muscle force generation is lower at midstance compared to other points during stance phase, particularly later in stance phase. Collectively this suggests that future research on lower limb muscle forces should consider including other points in the gait cycle in addition to midstance.

## MATERIALS AND METHODS

### Participants

Ten healthy participants (five females, five males) were recruited for this study. All participants reported that they were free from lower-limb injuries. The sex, age, body mass, and stature of each participant are reported in Table 1. The University of Washington's Institutional Review Board approved all aspects of this study (IRB#: STUDY00001125) and all participants signed informed consent forms prior to data collection.

### Experimental protocol

Kinetic and kinematic data were measured using a 10-camera motion capture system (Qualisys, Sweden) and four force plates (Kistler, Switzerland) in the Amplifying Movement & Performance (AMP) laboratory of the University of Washington. Thirty infrared-reflective markers (5 mm hemispherical markers) were affixed to anatomical landmarks (Table S1) and used to track motion through the laboratory. Using their self-selected normal pace, participants walked unshod the length (10 m) of the gait laboratory five times. Participants were prompted to walk at their natural pace and to ignore the force plates by focusing their attention on a distant wall. Participants walked in a straight path, and trials where a single foot contacted the surface of each force plate were retained. Trials during which both feet contacted a particular force plate, or a foot exceeded the force plate margins, were discarded and immediately redone. Participants were allowed several attempts prior to data collection to become familiar with the protocol. Marker data and the GRF were collected at 120 Hz and 1200 Hz, respectively. These data were filtered within the AnyBody modeling software (described below) using 10 Hz (GRF) or 5 Hz (marker data) cut-off frequencies with a 4th order, low pass zero-lag Butterworth filter. Calibration of the system yielded a limitation in its fidelity for marker data of 1 mm and force data of ±2.5N for the direction of travel (X), ±5N side (Y), and ±25N vertical (Z). In total, 50 trials (ten participants, five trials/participant) were collected. Data files for two trials for two subjects (four trials total) were subsequently found to be corrupt or contained significant marker dropout and were unusable, leaving 46 trials for analysis. Each trial included two complete stance phases (one left foot and one right foot) resulting in 92 total stance phases for analysis.

### Musculoskeletal model

We used the MoCap model from a repository (AnyBody Managed Model Repository AMMR v2.3.0), which is a validated, multi-trial, full-body, motion-capture-driven human gait model that is part of the commercially available AnyBody Modelling System (v.7.3, AnyBody Technology, Denmark), to calculate muscle forces in the lower limb (Damsgaard et al., 2006). This model has been used to assess human gait in numerous applications (De Pieri et al., 2018; Fischer et al., 2018; Dzialo et al., 2019; Obrębska et al., 2020). The MoCap model is assembled from a trunk (head, thorax, abdomen, etc.) and left and right lower limb components (TLEM 2.0; De Pieri et al., 2018). The lower limbs consist of the following segments: pelvis, thigh, patella, shank, talus, and foot segments. Each lower limb has six total degrees of freedom including all three rotations at the hip and one each at the knee (flexion/extension), ankle (plantarflexion/dorsiflexion), and subtalar (inversion/eversion) joints. The pelvis (relative to the ground) has six degrees of freedom, three translational and three rotational. Forty-one anatomical muscles are represented by 169 muscle elements (force actuators) in each model lower limb (e.g. gluteus medius is composed of 12 separate muscle element actuators).

### Musculoskeletal simulation

A standard and commonly used protocol was followed for the musculoskeletal simulation for each walking trial (De Pieri et al., 2018). First, the musculoskeletal model was scaled to match the individual (Andersen et al., 2009); this scaling includes mass and body segment dimensions while also optimizing marker locations. Not all markers were permitted to optimize in all coordinate directions; constraints for each marker are provided in Table S1. Following convergence of parameter optimization, we conducted an inverse kinematics analysis. Kinematic parameters (including displacement and linear and angular acceleration curves for the pelvis, thigh, and shank segments) were inspected for incongruity, such as sudden changes in segment accelerations, which can create variation in muscle activity (Fig. S1). Finally, we conducted the inverse dynamic analysis, which included allocating muscle activations to achieve the observed accelerations and GRF. The algorithm minimizes the cost function that is the sum of muscle activation values raised to the third power.

### Model-derived variables and data processing

We exported the force magnitudes from the 169 lower limb muscle element actuators for each limb during stance phase of walking. We determined the stance phase for each limb using GRF profiles and force plate contact detection output. All stance phases were resampled to 1% increments of the stance phase using a custom written routine (Matlab, USA). All stance phases for an individual (five trials, two stance phases=ten total stance phases, except for two subjects who had six total stance phases) were averaged to create a participant-specific muscle element profile. Muscle force magnitudes produced by elements within anatomically defined muscles (e.g. the 12 elements that represent gluteus medius) were summed as a measure of the demand on the musculoskeletal system and overall force application to the segments. Anatomically defined muscles with maximum muscle force that did not exceed 10% of body weight were excluded from analyses. Muscles that perform the same major function (e.g. hip flexion: iliacus and psoas; hip abduction: gluteus medius, gluteus minimus, and tensor fasciae latae) were summed for further analyses (functional muscle groups: Table 2). The sum of all muscle forces was also calculated at each time increment (Muscle Sum). For each functional muscle group and the Muscle Sum curve, we calculated an average force curve profile by averaging the participant-specific curves.

### Analysis

To determine which functional muscle group force curves demonstrated the highest consistency in the shape, we calculated all pairwise correlations between the ten participants based on their 101 time increment force values. For instance, given the ten participant-specific average force curves for the hip flexor functional muscle group, we calculated the correlation between each possible pair of participants using their respective force values for this functional muscle group. This procedure resulted in 45 unique correlation values for each functional muscle group. We calculated the average and standard deviation of the correlations within each functional muscle group. For muscles that demonstrate a consistent pattern, the average correlation should be relatively high, while curves with substantial variation in shape should have lower correlations. For each participant, five gait events were identified: initial contact; breaking peak in GRF; GRF minimum around midstance; propulsive peak in GRF; toe-off. Muscle forces were extracted at each gait event and expressed as a percentage of the sum of all muscle forces (Muscle Sum).

## Supplementary Material

Supplementary information
