# Supplementary material for: Muscle forces and the demands of human walking
Source: Biol Open. 2021 Jul 19;10(7):bio058595. doi: 10.1242/bio.058595 (PMC8325943; doi:10.1242/bio.058595)
Supplement: Supplementary information [file biolopen-10-058595-s1.pdf]

Table S1. Marker Names / Abbreviations in Model: Parent body segment. Anatomical description of the marker location. Coordinate directions optimized during parameter identification. If empty, all coordinate directions fixed.

Marker Name	Parent Segment	Anatomical Description	Optimized Coordinate Direction
Glabella	Skull	Glabella	
LAcrom, RAcrom	Thorax	Left and right acromion of clavicles	X
LASIS, RASIS	Pelvis	Left and right anterior superior	
LCrest, RCrest	Pelvis	Left and right iliac crest, most laterally projecting point	X,Y,Z
LILB	Pelvis	Lower back plate placed over junction of sacrum and lower lumbar	Y,Z
LAST, RAST	Thigh	Left and right anterior, superior position on thigh plate	X,Y,Z
LAIT, RAIT	Thigh	Left and right anterior, inferior position on thigh plate	X,Y,Z
LPIT, RPIT	Thigh	Left and right posterior, inferior position on thigh plate	X,Y,Z
LLatTib, RLatTib	Shank	Most laterally projecting point on left and right tibial plateaus	X,Z
LTibTub, LTibTub	Shank	Left and right tibial tuberosities [most anteriorly projecting point]	X,Y,Z
LMedTib, RMedTib	Shank	Most medially projecting point on left and right tibial plateaus	X,Z
LLatMal, RLatMal	Shank	Most laterally projecting point on left and right lateral malleoli	X,Y,Z
LMedMal, RMedMal	Shank	Most laterally projecting point on left and right lateral malleoli	Z
LMT5, RMT5	Foot	Left and right 5th metatarsal head	
LMT1, RMT1	Foot	Left and right 1st metatarsal head	
LHeel, RHeel	Foot	Most posteriorly projecting point on left and right calcanei	Y

Biology Open • Supplementary information

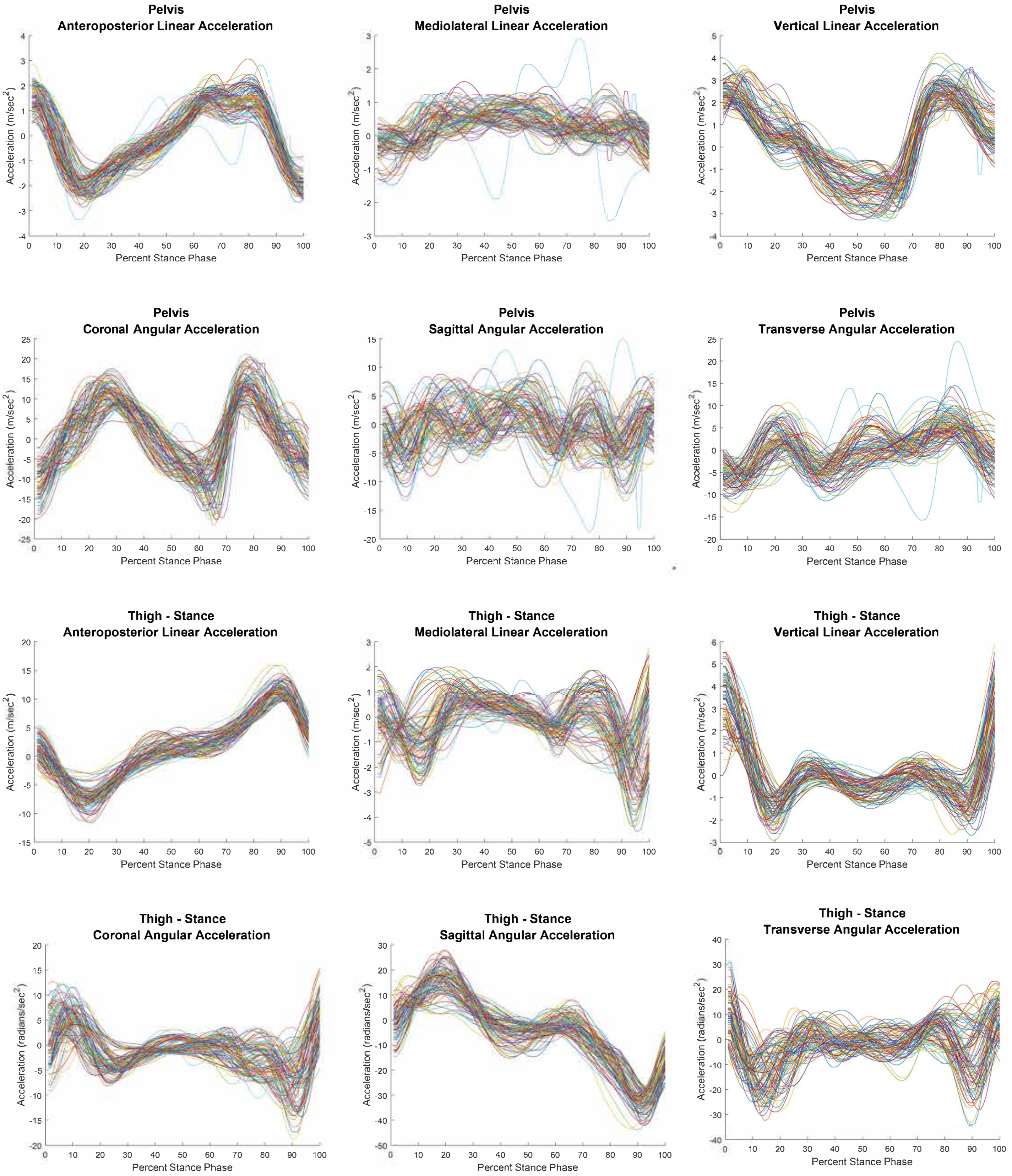

Biology Open • Supplementary information

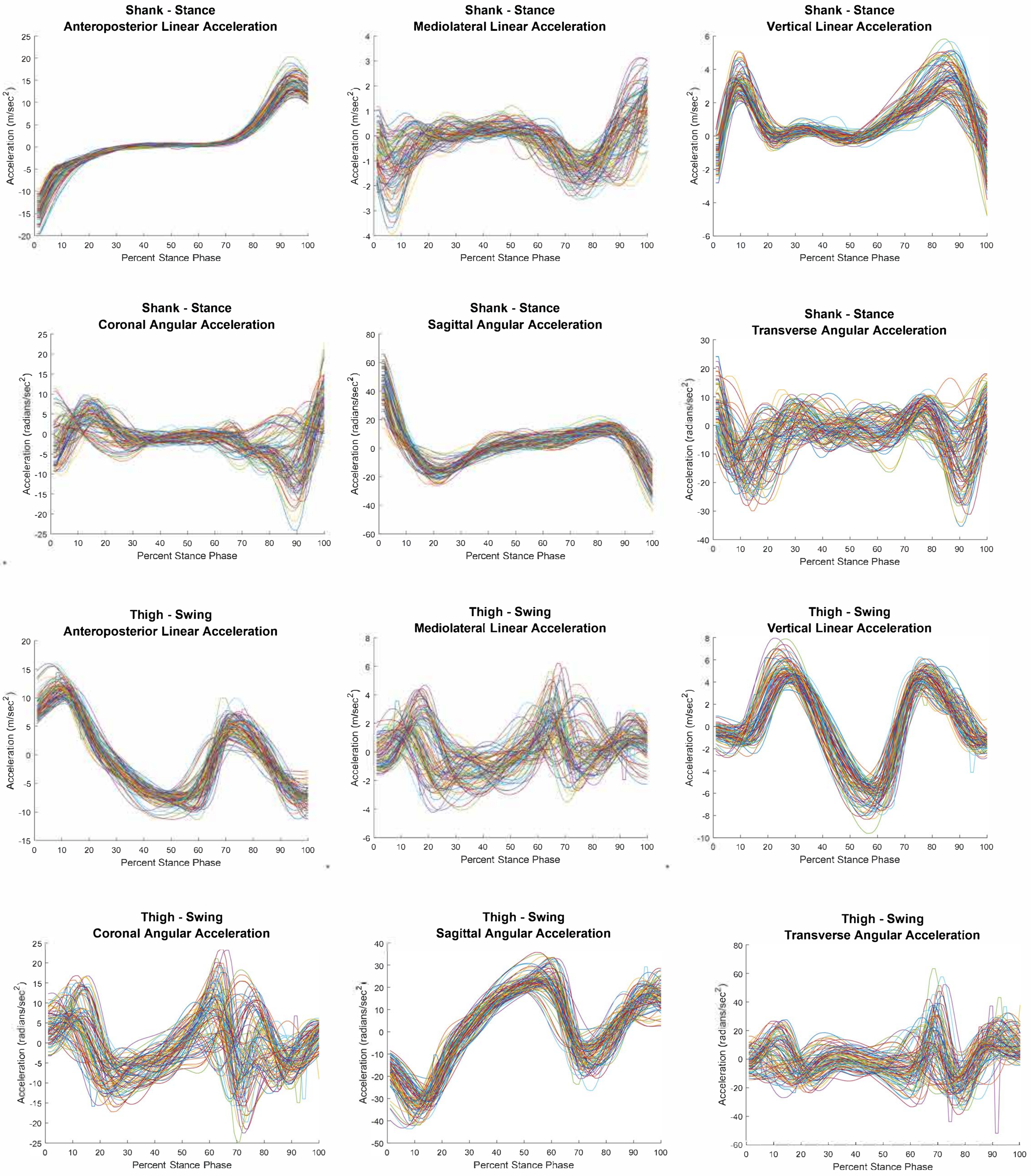

Biology Open • Supplementary information

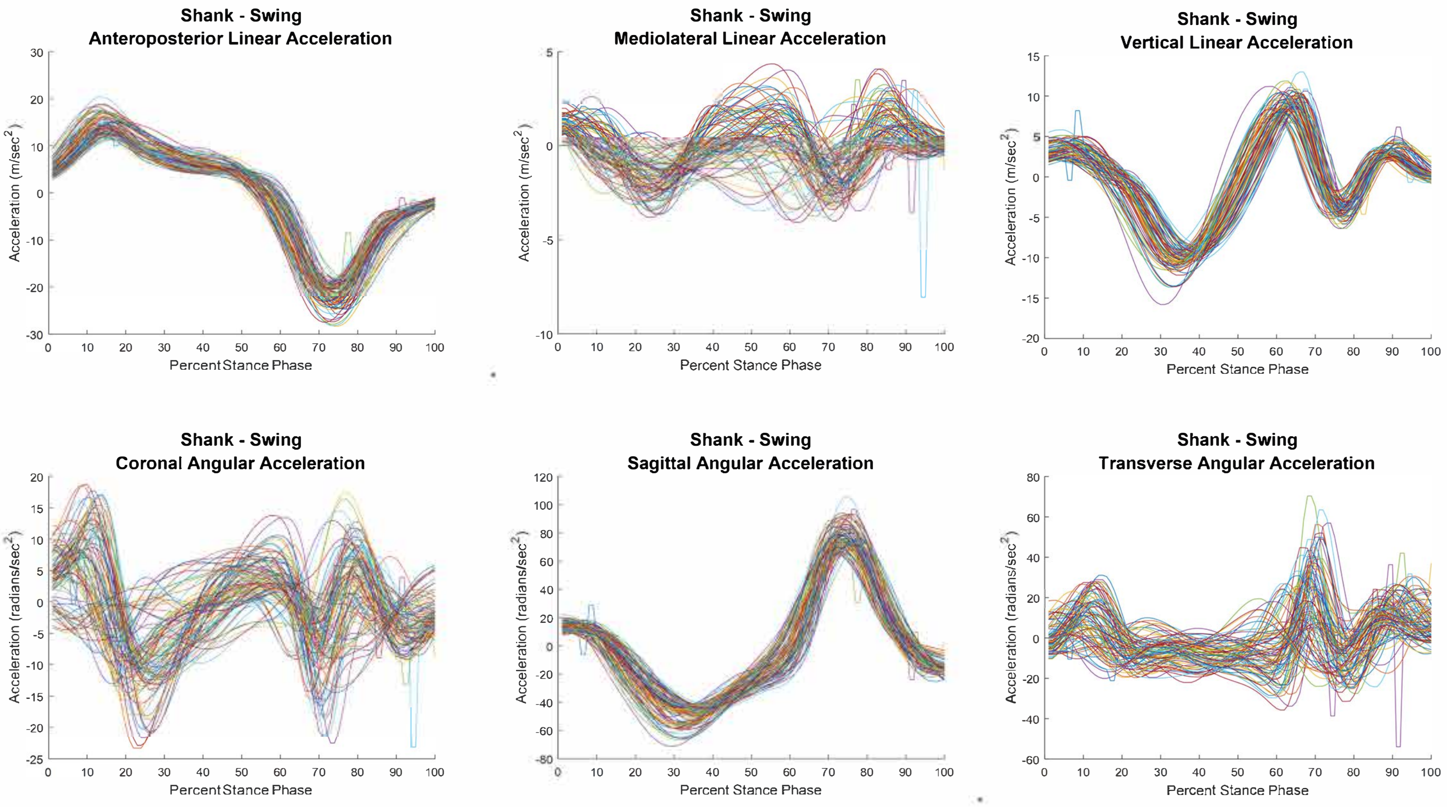

Figure S1. Segment Linear and Anguar Acceeratons fo Each Wakng Tia Stance Phase (n=92)

Biology Open • Supplementary information
